# Level-biases in estimated breeding values due to the use of different SNP panels over time in ssGBLUP

**DOI:** 10.1186/s12711-019-0517-z

**Published:** 2019-12-16

**Authors:** Øyvind Nordbø, Arne B. Gjuvsland, Leiv Sigbjørn Eikje, Theo Meuwissen

**Affiliations:** 1 GENO SA, Storhamargata 44, 2317 Hamar, Norway; 2 Norsvin R&D, Storhamargata 44, 2317 Hamar, Norway; 3 Norwegian University of Life Sciences, Postboks 5003 NMBU, 1432 Ås, Norway

## Abstract

**Background:**

The main aim of single-step genomic predictions was to facilitate optimal selection in populations consisting of both genotyped and non-genotyped individuals. However, in spite of intensive research, biases still occur, which make it difficult to perform optimal selection across groups of animals. The objective of this study was to investigate whether incomplete genotype datasets with errors could be a potential source of level-bias between genotyped and non-genotyped animals and between animals genotyped on different single nucleotide polymorphism (SNP) panels in single-step genomic predictions.

**Results:**

Incomplete and erroneous genotypes of young animals caused biases in breeding values between groups of animals. Systematic noise or missing data for less than 1% of the SNPs in the genotype data had substantial effects on the differences in breeding values between genotyped and non-genotyped animals, and between animals genotyped on different chips. The breeding values of young genotyped individuals were biased upward, and the magnitude was up to 0.8 genetic standard deviations, compared with breeding values of non-genotyped individuals. Similarly, the magnitude of a small value added to the diagonal of the genomic relationship matrix affected the level of average breeding values between groups of genotyped and non-genotyped animals. Cross-validation accuracies and regression coefficients were not sensitive to these factors.

**Conclusions:**

Because, historically, different SNP chips have been used for genotyping different parts of a population, fine-tuning of imputation within and across SNP chips and handling of missing genotypes are crucial for reducing bias. Although all the SNPs used for estimating breeding values are present on the chip used for genotyping young animals, incompleteness and some genotype errors might lead to level-biases in breeding values.

## Background

Bias in single-step genomic predictions for dairy cattle has been debated within scientific communities for several years [1–3], and these biases have hindered the breeding organisations from adopting single-step genomic best linear unbiased prediction (GBLUP) approaches. Bias is a general term, and for practical use in breeding programmes, it is useful to differentiate between at least two types of bias: (1) inflation of genomic breeding values, which causes an enhanced spread of the breeding value estimates; and (2) level-bias of breeding values, which influences the predicted genetic levels for groups of animals (e.g. overprediction of genotyped animals vs. non-genotyped animals). Since many dairy breeding organisations tend to use only young bulls without daughter information in their breeding program, the importance of the inflation becomes less problematic for genetic progress. On the contrary, a prominent level-bias can have important effects for two reasons: (1) pre-selection (selection of the calves that should be genotyped) is based on the mid-parent mean, and a prominent level-bias would affect the selection based on the genotype status of the mother; (2) if the herd consists of both genotyped and ungenotyped animals, level-bias would affect the selection accuracy within the herd.

In single-step genomic predictions (ssGBLUP), breeding values for the selection of both genotyped and non-genotyped animals are calculated simultaneously with all the information included [4, 5]. This makes it possible to perform all the selection within the breeding program based on one set of breeding values. To perform an optimal selection, it is crucial that predicted breeding values are on the same scale whether the animal is genotyped or not.

Often, selection of young animals to be genotyped (before the final selection into the artificial insemination (AI) and embryo transfer programs) is based on the parent-mean estimated breeding value (EBV) of all the calves in the entire population. Most AI bulls are genotyped; however, the cow population consists of both genotyped and non-genotyped animals. In the presence of level-bias, where genotyped cows that on average have higher breeding values than non-genotyped cows, sons and daughters of genotyped cows will mainly have higher parent average EBV than descendants of non-genotyped cows, and this leads to incorrect ranking and reduced genetic progress.

In 2012, genomic predictions for all traits were implemented for the pre-selection of Norwegian Red young bulls using a SNP-BLUP method [6]. Selection candidates were genotyped on the same SNP panel as most of the reference population. In 2016, SNP-BLUP was replaced by single-step GBLUP [4, 5] because of the higher accuracy of EBV. This was followed by intensive genotyping of cows, using another SNP panel. All the SNPs used for genomic predictions were present on the new panel, and no imputation was performed when new genotypes from the new chip were added. After some months in operation, it was observed that breeding values of different groups of animals tended to drift. Young genotyped animals received on average higher EBV than before genotyping, and in addition, animals genotyped on one chip were predicted to have a higher genetic level than animals genotyped on other SNP chips, and this deviation was larger than that explained by genetic trend.

In this study, we investigated several potential sources of the aforementioned biases. We investigated whether incomplete imputation of missing SNPs or erroneous SNP-data could be sources of bias between genotyped and non-genotyped animals and between animals genotyped on different SNP panels in ssGBLUP. We also examined how the addition of a small value to the diagonal of the genomic relationship matrix, which was previously often used to make it non-singular, affects level-bias between genotyped and non-genotyped animals.

## Methods

### Population

Norwegian Red (NR) is a synthetic dairy breed, which has been selected for a broad breeding objective. Historically, it was based on a cross between traditional Norwegian breeds, in combination with imported genetics mainly from Finnish Ayrshire and Swedish Red (from 1960 to present), but also some Holstein/Friesian in the 1960s and 1970s. Currently, about 5% of the NR originates from Holstein (Morten Svendsen, personal communication). Back in the 1950s and 1960s, Norwegian Reds were mainly selected for production and functionality. Later, other traits were included in the breeding goal, i.e. fertility in the early 1970s and health in the late 1970s. Currently, health and fertility traits constitute one third of the breeding goal and the rest is divided between functional traits (1/3), which include both conformation traits, milkability, temperament and polledness, and production traits (1/3), which include both milk and beef production). The population includes ~ 210,000 dairy cows, with an effective population size of about 240 [7].

### Phenotypes and statistical model

Phenotypes on 305-day lactation yields for kg of milk were taken from the routine evaluations consisting of 7,519,418 records from the 1st to 3rd lactation on 3,647,173 Norwegian Red cows, with lactation data from 1979 and onwards. The pedigree was traced back to 25 generations and comprised 4,650,339 animals. Missing pedigree data were grouped by year of birth and by the following classes: the missing parent is the missing AI sire, or the missing farm bull or the missing dam. This resulted in 114 groups. The dataset was analysed in MiX99 [8] by a single-trait repeatability animal model with a heritability, $h^{2}$ equal to 0.26, and a value of repeatability equal to 0.52, which are the values used in routine evaluations:

$${\mathbf{y}} = \mu + {\mathbf{Tm}} + {\mathbf{Fa}} + {\mathbf{Kd}} + {\mathbf{Xh}} + {\mathbf{ZQ}}\widehat{{\mathbf{g}}} + {\mathbf{Pp}} + {\mathbf{Z}}\widehat{{\mathbf{u}}} + {\mathbf{e}},$$

where ${\mathbf{y}}$ is the vector of estimated accumulated 305 days yields/lactation produced by the cow; $\mu$ is the intercept; ${\mathbf{m}}$ is a vector of fixed month $\times$ year effects with the design matrix ${\mathbf{T}}$; ${\mathbf{a}}$ is a vector of fixed age $\times$ lactation number effects with the design matrix ${\mathbf{F}}$, (age was measured in months); ${\mathbf{d}}$ is a vector of fixed effects of days open $\times$ lactation number with the design matrix ${\mathbf{K}}$, (days open is grouped into 10-days intervals up till 159 days); ${\mathbf{h}}$ is a vector of random herd $\times$ year effects with the design matrix ${\mathbf{X}}$; $\widehat{{\mathbf{g}}}$ is the vector of genetic group regression effects with the design matrix ${\mathbf{Q}}$ of genetic group contributions; ${\mathbf{p}}$ is a vector of random permanent environmental effects with the design matrix ${\mathbf{P}}$; $\widehat{{\mathbf{u}}}$ is a vector of random animal effects with the design matrix ${\mathbf{Z}}$; and ${\mathbf{e}}$ is a vector of random errors. All random effects are assumed to be independently distributed, with the exception of $\widehat{{\mathbf{u}}}$ which has variance ${\text{Var}}\left( {\mathbf{u}} \right) = {\mathbf{H}}\sigma_{{\mathbf{u}}}^{2}$, where ${\mathbf{H}}$ denotes the combined genomic and pedigree-based relationship matrix that is varied as described in the next section. Genetic groups were included in the model as fixed regressions where the genetic group contributions of each animal in ${\mathbf{Q}}$ were calculated first with RelaX2 [9]. Then, after solving the mixed model equations in MiX99, the regression coefficients $\widehat{{\mathbf{g}}}$, were multiplied with the genetic group matrix ${\mathbf{Q}}$ (which included all the animals in the pedigree) and then added to the animal effects ($\widehat{{\mathbf{u}}}$) to give the final breeding values: ${\text{GEBV}} = \widehat{{\mathbf{u}}} + {\mathbf{Q}}\widehat{{\mathbf{g}}}.$

### Genotype data and genomic relationships

**Fig. 1:**
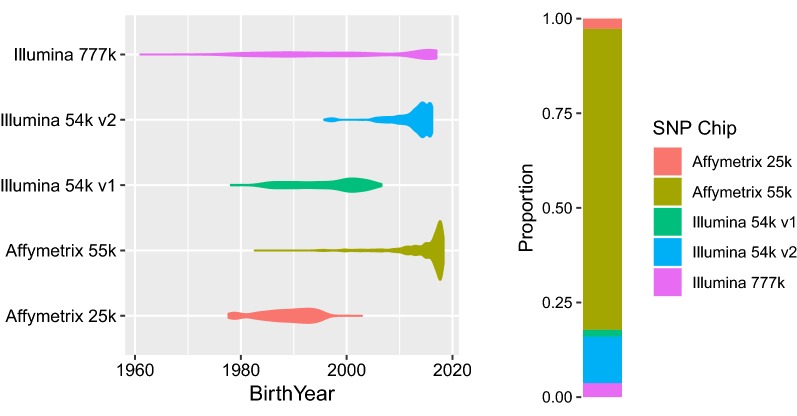
Birth year and proportion of animals genotyped on different SNP chips

Genomic data consisted of 40,174 Norwegian Red animals, genotyped on different platforms: a customized Affymetrix 55 k SNP chip (Affymetrix, Santa Clara) (31,947 samples), Illumina 54 k v1, BovineSNP50 BeadChip (Illumina, San Diego) (726 samples), Illumina 54 k v2, BovineSNP50 BeadChip (Illumina, San Diego) (4943 samples), Illumina 777 k, BovineHD Genotyping BeadChip (Illumina, San Diego) (1487 samples) and Affymetrix 25 k (Affymetrix, Santa Clara), (1071 samples). Most progeny-tested bulls are genotyped on the Illumina chips and the Affymetrix 25 k chip, whereas in the last few years most of the cows and selection candidates have been genotyped on the Affymetrix 55 k SNP chip (Fig. 1).

The following parameters were used to filter the SNPs within a chip before imputation: a minor allele frequency (MAF) higher than 0.01, a SNP call rate $\ge$ 0.9, and a Hardy–Weinberg equilibrium exact test *p* value $>$ 1e–7. In order to reduce the biases observed in the routine breeding value estimations described in the "Background" section, genotype data were imputed with Flmpute [10] to a subset of the Illumina 777 k chip for which all SNPs segregated in the population with few Mendelian inconsistencies. To make the selection of SNPs for genomic predictions as uniform as possible across the genome, we performed linkage disequilibrium (LD) pruning by using the PLINK software [11, 12]. A 50-SNP window size was set, and five SNPs was used to shift the window at each step. Pairs of variants in the current window with a squared correlation greater than the $r^{2}$ threshold of 0.5 were pruned from the window until no such pairs remained. After this step, 109 k SNPs remained for the genomic predictions.

SNP data were used to calculate genomic relationships and to build the combined inverse pedigree and genomic relationship matrix ${\mathbf{H}}^{ - 1}$ [4, 5] using the HGINV program [13]:

$${\mathbf{H}}^{ - 1} = \left[ {\begin{array}{*{20}c} 0 & 0 \\ 0 & {{\mathbf{G}}_{\text{w}}^{ - 1} - {\mathbf{A}}_{22}^{ - 1} } \\ \end{array} } \right] + {\mathbf{A}}^{ - 1} ,$$

where ${\mathbf{A}}_{22}$ is the sub-matrix of the pedigree-based relationship matrix (${\mathbf{A}}$) for genotyped animals. Since Norwegian Reds’ are an open population, estimation of the base allele frequencies is not straightforward. To overcome this problem and to avoid that imported genetics appeared extremely inbred, we used equal allele frequencies (0.5) for all SNPs in the construction of the genomic relationship matrix ${\mathbf{G}}$ [14]. ${\mathbf{G}}$ was then scaled by multiplying a parameter to all matrix elements to make the average diagonal elements equal to 1 [15]. Let ${\mathbf{M}}$ denote the ($m*n$) marker matrix, with $m$ individuals and $n$ markers containing elements equal to − 1, 0 and 1 for the homozygote, heterozygote and the other homozygote, respectively. Using Method 1 from [16] with allele frequencies of 0.5 and scaling the matrix to have average diagonal elements equal to 1, the scaled genomic relationship matrix can be written as ${\mathbf{G}} = \frac{{\mathbf{m}}}{{{\mathbf{trace}}\left( {{\mathbf{MM}}^{{\mathbf{T}}} } \right)}}{\mathbf{MM}}^{{\mathbf{T}}}$. Then, the additive pedigree relationship matrix was weighted by 10% to account for genetic effects that are not captured by the SNPs. In addition, we also investigated the effect of adding a small value (${\text{Diag}}$) to the diagonal of the raw ${\mathbf{G}}$ matrix [13, 17], which became ${\mathbf{G}}{\text{w}} = 0.9 *\left( {{\mathbf{G}} + {\mathbf{I}}*{\text{Diag}}} \right) + 0.1 *{\mathbf{A}}_{22} .$ The effect of changing the ${\text{Diag}}$ parameter was investigated for the alternative scenarios D-2 to D-4 in Table 1, while for the other scenarios, this parameter was set to zero.

**Table 1:** Description of the scenarios used to mimic different types of genotype errors and parameters set

Scenario	Diag^a^	Further description
1	0	1000 missing random SNPs for each Affy^b^ animal
2	0	1000 common random missing SNPs for Affy animals
3	0	1000 common random SNP genotypes randomly changed for Affy animals, equal probability for all genotypes
4	0	1000 common random SNP genotypes randomly changed for Affy animals, probability based on Hardy–Weinberg genotype frequency
BaseLine	0	
D-2	10^−2^	
D-3	10^−3^	
D-4	10^−4^	

^a^Parameter added to the diagonal of the genomic relationship matrix to make it invertible

^b^Animal genotyped on the customized Affymetrix 55 k SNP chip

To investigate various aspects of imputation of missing data and discrepancy between SNP panels, we made some permutations of the SNP data to mimic different scenarios before running the HGINV program with the same parameters as mentioned above. In raw genotype data, noise or missing data occur for nearly 1% of the SNPs, and here 1000 of the 109 k SNPs were permuted. The HGINV program imputes missing genotypes by inserting the most common genotype. Scenario 1 was motivated by the situation where there is a proportion of the genotypes that are not imputed before going into HGINV, but there is no systematic missingness among the SNPs (Table 1). This was performed by setting 1000 random SNPs for each animal genotyped on the Affymetrix 55 k SNP chip to missing (a different set of 1000 SNPs for each of the animals). To study the effect of systematic missingness among SNPs for non-imputed genotypes, we created Scenario 2, in which, 1000 common random SNPs were set to missing for all animals genotyped on the Affymetrix 55 k chip (‘common’ denotes that the set of SNPs was the same for all these animals). Scenario 3 was motivated by our experience that some systematic genotyping errors exist. Some SNPs work well on one chip and poorly on others. This was modelled by removing data on a set of 1000 common random SNPs for all the animals genotyped on the Affymetrix 55 k chip and inserting random genotypes from a uniform multinomial distribution on those 1000 SNPs instead (equal probability of inserting 0, 1 or 2). Finally, we developed Scenario 4, to investigate alternative handling of missing data. In this scenario, the genotypes of the common 1000 random SNPs among the animals genotyped on the Affymetrix 55 k chip were removed and replaced by one of the three possible genotypes according to the distribution of their probabilities (based on Hardy–Weinberg equilibrium frequencies, which were estimated based on the animals genotyped on the other SNP chips).

To investigate how sensitive the predictions are to the set of random values used for creating missingness and errors, each of the scenarios 1 to 4 was replicated ten times and standard errors for the different measures were calculated based on these replicates.

### Measurement of level-bias

To quantify level-bias between genotyped and non-genotyped animals for the different scenarios, we masked the genotypes of 2000 young animals without progeny or phenotypes, genotyped with the Affymetrix 55 k chip. Then, we ran single-step genomic prediction and compared the breeding values of these animals ($GEBV_{out}$) with those when their genotypes were included ($GEBV_{in}$). The average change in breeding value of these animals divided by the genetic standard deviation of the trait, $\sigma_{g}$, quantified level-bias in the model.

$${\text{LevelBias}} = \frac{{{\text{mean}}\left( {GEBV_{in} - GEBV_{out} } \right)}}{{\sigma_{g} }}.$$

To measure level-bias between animals genotyped on the Affymetrix 55 k chip vs. the other panels, we analysed only the genotyped animals that were born between 31.12.2013 and 01.01.2017. The reason is that we wanted to remove the effect of genetic trend from the dataset. We then investigated the mean level of the breeding values from the different scenarios and compared them with those from pedigree-based BLUP.

### Measurement of accuracy and inflation

To estimate the accuracy and inflation of genomic estimated breeding values (GEBV), we removed the phenotypes of the 4000 youngest genotyped cows with milk records and all the data on their descendants. Then, we estimated the breeding values and correlated them against the yield deviations (YD) on the same 4000 animals. The yield deviations were calculated as the sum of GEBV plus the residual connected to each record from the full dataset from MiX99 [8]. Since some of the validation cows had more than one lactation record, the number of YD (6285) was a little larger than the number of animals (4000). Animals with more than one record had their GEBV repeated in the dataset before estimating the correlation and performing a linear regression. Accuracy $r$ was then measured as:

$$r = \frac{{{\text{cor}}\left( {GEBV,YD} \right)}}{{\sqrt {h^{2} } }}.$$

Inflation was estimated as the regression coefficient $\beta$ from the linear regression with YD as a response variable and the GEBV as an explanatory variable.

## Results

Level-biases for the 2000 young animals in the different scenarios are in Table 2, which shows that the magnitude of level-bias depends on the noise in the genotype data and, to some extent, on the parameter added to the diagonal. The accuracy and inflation (regression coefficient) are much less sensitive to these permutations.

**Table 2:** Accuracy and regression coefficients of genomic predictions as well as a measure of level-bias between genotyped vs. non-genotyped animals

Scenario^a^	Accuracy	Regression coefficient^b^	Level-bias^c^
1	0.871 (0.000)	1.135 (0.000)	0.151 (0.002)
2	0.869 (0.000)	1.127 (0.001)	0.768 (0.002)
3	0.864 (0.000)	1.120 (0.001)	0.812 (0.002)
4	0.870 (0.000)	1.128 (0.001)	0.172 (0.002)
BaseLine	0.873	1.130	0.086
D-2	0.872	1.096	0.193
D-3	0.873	1.128	0.104
D-4	0.873	1.130	0.096

For Scenarios 1 to 4, the standard errors due to the random sampling effect of SNPs are in parentheses

^a^Scenarios described in Table 1

^b^Regression coefficient $\beta$ from the linear regression with YD as a response variable and the GEBV as an explanatory variable

^c^Level-bias is measured in genetic standard deviations

**Fig. 2:**
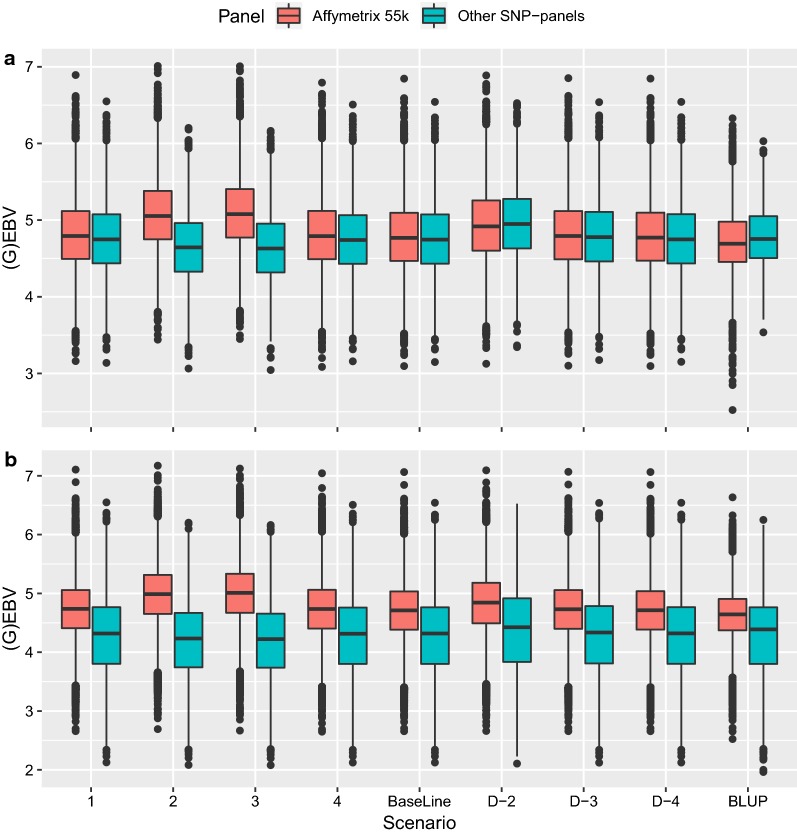
Box plot of breeding values of **a** genotyped animals born between 2013 and 2017 and **b** all genotyped animals in the different scenarios

For all scenarios, the predicted GEBV for animals born after 2013 and genotyped on the Affymetrix 55 k chip were higher than those predicted with BLUP (Fig. 2a). The BLUP-EBV of the group of animals genotyped on the Affymetrix 55 k chip were slightly lower than those for the animals genotyped on other SNP panels. In contrast, for some of the other scenarios (particularly, Scenarios 2 and 3), this relationship was reverted. The only scenario, for which the GEBV between animals genotyped on the Affymetrix 55 k chip vs. other SNP panels reached the same values as with BLUP, was Scenario D-2. However, in this scenario, both groups of genotyped animals (on the Affymetrix 55 k chip and other SNP panels) had increased GEBV compared to the BLUP-EBV. Comparison of the breeding values for all genotyped animals (Fig. 2b) showed that the breeding values for animals genotyped on the Affymetrix 55 chip are on average higher than those for animals genotyped on other SNP chips.

## Discussion

In this study, we demonstrated how genotyping errors and missing values in genotype data affect level-bias in single-step genomic predictions. Noise or missing data for nearly 1% of the SNPs in the genotype data are in the same range of those observed with raw genotype data, and they affected both the level of breeding values between genotyped and non-genotyped animals, and between genotyped animals genotyped on different chips.

Scenarios 2 and 3 gave the highest level-bias (Table 2). These scenarios have in common that the introduced genotype missingness/errors resulted in systematic differences in allele frequencies between genotypes obtained with Affymetrix 55 k and the other SNP chips and seemed to cause bias between genotyped vs. non-genotyped animals as well as between animals genotyped on different SNP panels (Fig. 2a). On the one hand, the animals belonging to the group genotyped with the other SNP panels mainly consist of progeny-tested bulls born over a period from 1980 to 2014. On the other hand, the Affymetrix genotype data consist mostly of cows, heifers, calves and selection candidates that were born within the last 5 years and have a higher average genetic level than the animals belonging to the group of animals genotyped on other SNP panels (Fig. 2b). When allele frequency is changed due to missingness and errors in the raw genotype data, these SNPs will become erroneously associated with the genetic improvement that occurred between these two groups of animals. New animals with added raw-genotypes will receive biased breeding values because their genotype will carry some of the same missingness and noisy SNPs as the other animals genotyped on the same chip. This shows that filtering within chips (based on Hardy–Weinberg equilibrium and missingness), in addition to imputation in routine single-step genomic evaluations, is crucial to reduce level-bias.

If routine imputation is not possible due to computational costs, inserting the most common genotype or a uniformly random genotype is an extremely poor choice. Instead, picking a random genotype based on the observed allele frequencies is better, as illustrated in Scenario 4. Another approach is to use genotype probabilities instead of poorly imputed genotypes, but the software for building genomic relationship matrices that were developed for large-scale genotype datasets are generally not able to handle genotype probabilities (such as [13, 17]).

The value of the parameter added to the diagonal of the ${\mathbf{G}}$ matrix also influenced level-bias, but to a much smaller extent than the permutations mentioned above. The permutations in the diag-parameter mainly affected level-bias between genotyped and non-genotyped animals. A value of 0.01 (such as in e.g. [18]) added to the diagonal elements of the genomic relationship matrix more than doubled level-bias between genotyped and non-genotyped animals, compared with the BaseLine scenario (see Table 2). Instead of adding values to the diagonal values, a better idea is to put some weight on the ${\mathbf{A}}$-matrix which makes the genomic relationship matrix invertible [4], and also, results in improved prediction accuracy [19].

In addition, the scenarios with non-systematic random missing values among the animals genotyped on the Affymetrix 55 k chip (Scenario 1), and the scenario in which systematic missingness was replaced by a random number drawn from the observed Hardy–Weinberg genotype distribution (Scenario 4), gave the same level-bias between genotyped and non-genotyped animals. These two scenarios do not necessarily create systematic differences in allele frequencies between genotypes obtained with Affymetrix 55 k and other SNP chips. Thus, they do not affect level-bias between the groups of animals genotyped on the Affymetrix 55 k chip vs. other SNP chips. These scenarios resulted in values of level-bias that were similar to those obtained when adding a relatively large number to the diagonal of the genomic relationship matrix.

Cross-validation of different models is a valuable tool to optimize a methodology, but not necessarily the best tool to uncover level-bias. Many studies quantify the performance of a model by measuring the accuracy and regression coefficients only, but these measures are relatively insensitive to the values of the input parameters [1, 19]. Normally a validation set is selected to be a set of genotyped animals born within a brief time interval, but such validations do not capture the level-bias described in this paper. Level-bias seems to be much more sensitive to changes in parameters than regression coefficients and accuracy, which are mainly measured when comparing models. Since level-bias affects the average level of EBV for groups of animals, the average ranking within the group might be correct, but the average level across the groups might be wrong. To quantify level-bias, validation sets should contain enough animals from the relevant groups. Alternatively, one could do comparisons as shown here, by removing some of the animals and comparing the average change in their estimated breeding values when including their genotype data.

Several attempts have been done to reduce both bias and inflation by introducing and optimizing parameters with more or less biological meaning [20] and by introducing variables that could represent the selection history [1, 2]. In practice with noisy genotype data, a lot of the observed bias in single-step genomic predictions could be reduced without inserting any new parameters or variables. However, since single-step genomic predictions depend on many assumptions and parameters and determining which parameters should be tuned to get rid of the bias is not straightforward. Because historically different SNP chips have been used for genotyping different parts of the population, fine-tuning of imputation across SNP chips and handling of missing genotypes is crucial. This does not mean that the work performed to harmonize ${\mathbf{A}}$ and ${\mathbf{G}}$ and account for selection history [1–3] is not important. In a subsequent paper, we shall investigate how such methods could further reduce level-bias in our data.

Contrary to other studies [19, 21, 22], the GEBV of production traits do not seem to be inflated, but rather show too little variance compared to yield deviations. This could be due to an underestimated genetic variance. Legarra [23] showed that genetic variance estimates depend on the base population assumed when estimating the relationship matrix. The genetic variance in the current population is $D_{k} \sigma_{g}^{2}$, where $D_{k}$ equals the average of the self-relationships minus the average of all the elements of the relationship matrix. In our case, $D_{k} = 0.63$ and the genetic variance was not estimated using this relationship matrix, which may explain why we effectively used a genetic variance that was too small.

The level-bias that we obtained in the ssGBLUP evaluations for Norwegian Red was probably due to a combination of various sources: lack of imputation of new genotypes, which led to partly systematic and partly random missingness among young genotyped animals, and in addition, erroneous genotypes. In addition, a diag parameter equal to 0.01 also contributed to level-bias. A lot of effort was done to harmonize the genomic and the pedigree part of the ${\mathbf{H}}^{ - 1}$ matrix [3, 4, 14], without success. However, removal of SNPs with a large amount of Mendelian inconsistencies across SNP chips, thorough imputation and decrease of the diag parameter, removed the most severe source of bias.

## Conclusions

We showed that incomplete handling of erroneous/missing genotypes of young animals resulted in high level-bias in breeding values in ssGBLUP. Both the level of breeding values between genotyped and non-genotyped animals and between animals genotyped on different SNP chips were affected. Similarly, the magnitude of a small value added to the diagonal of the genomic relationship matrix, to make it invertible, affected the level of breeding values between genotyped and non-genotyped animals. The accuracy and regression coefficient from standard cross-validations were not sensitive to erroneous/missing genotype data. Hence, we suggest that the average change in breeding value after the animals are genotyped, should be included as a standard quality measure of ssGBLUP models.

## Data Availability

The data that support the findings of this study are available from Geno SA, but restrictions apply to the availability of these data, which were used under license for the current study, and thus are not publicly available. However, data are available from the authors upon reasonable request and with permission of Geno SA.
